# Guideline Adherence of β-blocker Initiating Dose and its Consequence in Hospitalized Patients With Heart Failure With Reduced Ejection Fraction

**DOI:** 10.3389/fphar.2021.770239

**Published:** 2021-11-16

**Authors:** Yiling Zhou, Yuping Zeng, Si Wang, Nan Li, Miye Wang, Ify R. Mordi, Yan Ren, Youlian Zhou, Ye Zhu, Haoming Tian, Xin Sun, Xiaoping Chen, Zhenmei An, Chim C. Lang, Sheyu Li

**Affiliations:** ^1^ Department of Endocrinology and Metabolism, West China Hospital, Sichuan University, Chengdu, China; ^2^ Department of Laboratory Medicine, West China Hospital, Sichuan University, Chengdu, China; ^3^ Department of Cardiology, West China Hospital, Sichuan University, Chengdu, China; ^4^ The Informatic Center, West China Hospital, Sichuan University, Chengdu, China; ^5^ Division of Molecular and Clinical Medicine, School of Medicine, University of Dundee, Scotland, United Kingdom; ^6^ Cochrane China Center, MAGIC China Center, Chinese Evidence-Based Medicine Center, West China Hospital, Sichuan University, Chengdu, China; ^7^ Engineering Research Center of Medical Information Technology, Ministry of Education, West China Hospital, Sichuan University, Chengdu, China; ^8^ Division of Population Health and Genomics, School of Medicine, University of Dundee, Scotland, United Kingdom

**Keywords:** inverse probability weighting, heart failure, heart failure with reduced ejection fraction, hospitalization, beta blocker, adverse events < patient safety, guideline adherence [MeSH term], electronic medical records

## Abstract

**Background:** We aim to investigate the guideline adherence of β-blocker (BB) initiating dose in Chinese hospitalized patients with heart failure with reduced ejection fraction (HFrEF) and whether the adherence affected the in-hospital outcomes.

**Methods:** This was a retrospective study of patients hospitalized with HFrEF who had initiated BBs during their hospitalization. We defined adherence to clinical practice guidelines as initiating BB with standard dose and non-adherence to guidelines if otherwise, and examined the association between adherence to guidelines and in-hospital BB-related adverse events. Subgroup analyses based on sex, age, coronary heart disease, and hypertension were performed.

**Results:** Among 1,104 patients with HFrEF initiating BBs during hospitalization (median length of hospitalization, 12 days), 304 (27.5%) patients received BB with non-adherent initiating dose. This non-adherence was related to a higher risk (hazard ratio [95% confidence interval]) of BB dose reduction or withdrawal (1.78 [1.42 to 2.22], *P* < 0.001), but not significantly associated with risks of profound bradycardia, hypotension, cardiogenic shock requiring intravenous inotropes, and severe bronchospasm requiring intravenous steroid during hospitalization.

**Conclusion:** This study identified that over a fourth of patients had received BBs with an initiating dose that was not adherent to guidelines in Chinese hospitalized patients with HFrEF, and this non-adherence was associated with BB dose reduction or withdrawal during hospitalization.

## Highlights

### What is Already Known About This Subject?

• Current treatment guidelines recommend a “start low, go slow” strategy when initiating β-blocker (BB) treatment for patients with heart failure with reduced ejection fraction (HFrEF).

### What Does This Study Add?

• A fourth of patients were initiated on a starting dose of BB that was not adherent to the guideline recommendation among Chinese hospitalized patients with HFrEF during hospitalization.
• Initiating BB with a higher dose was associated with dose reduction or withdrawal of the BB treatment.
• Guideline-recommended initiating dose of BB may fit the hospitalized scenario in patients with HFrEF.

## Introduction

Heart failure (HF) is a major and growing public health problem worldwide including in China (Liu et al., 2014; Bozkurt et al., 2021). The aging population and improved survival from myocardial infarction have led to the continued increase in HF prevalence in divisions of cardiology and internal medicine, as well as the resulting high burden of hospitalizations and health care costs among individuals with HF (Ziaeian and Fonarow, 2016). HF with reduced ejection fraction (HFrEF), which is defined as left ventricular ejection factor (LVEF) <40%, approximately accounts for 50% of cases with HF (Maggioni et al., 2013; Murphy et al., 2020).

In the past 3 decades, dramatic advances have been made in the understanding of the pathophysiology of HF and the development of pharmacologic therapies that improve functional status and reduce hospitalizations and mortality for patients with HFrEF. These advances have led to guideline recommendations for the use of beta-blockers (BB), angiotensin-converting enzyme inhibitors (ACEI), or angiotensin receptor blockers (ARB), and mineralocorticoid receptor antagonists (MRA) in patients with symptomatic HFrEF (Teng et al., 2018).

Increasing evidence shows that insidious symptoms may be overlooked in people with early-stage HF in primary care until the emergency room visit due to acute exacerbation of the HF condition (Bottle et al., 2018). The majority of patients with HFrEF may therefore receive their first BB dose during their hospital stay rather than in the community setting following discharge (Smeets et al., 2016; Bottle et al., 2018). For these patients with incident HF hospitalization, the prescribing of HF medications at discharge including BBs has been used as an indicator of the quality of care (Gattis et al., 2004; Fonarow et al., 2007). It is, however, noteworthy that BB could be initiated at the time of presentation with an episode of acute decompensated HF.

Current clinical practice guidelines (CPGs) recommend a “start low, go slow” strategy of BB treatment, suggesting that BBs be started at low doses, not more than 1/8 of the target dose, and are slowly uptitrated over weeks or months (Chinese Society of Cardiology of Chinese Medical Association, 2014; Ponikowski et al., 2016). However, many clinicians argue for a higher starting dose of BB during hospitalization to avoid delay or possibly failure to uptitrate the BBs after discharge due to unfamiliar use of BBs in HF among general practitioners (GPs) in the community (Fuat et al., 2003; Hancock et al., 2014; Smeets et al., 2016). Many GPs continue to consider the initiation of BBs to be in the critical care pathway in the hospital (Ansari et al., 2003; Ventura, 2004; Bhatt et al., 2017).

Nevertheless, it is unclear what the practice of Chinese clinicians with initiating doses of BBs in patients with HFrEF in the real-world hospital setting is. The consequence of non-adherence to CPG recommendations is not known. We have conducted a population-based study to evaluate the proportion of patients who had been initiated on a starting dose of BBs that was non-adherent to CPG recommendations and explore the impact of non-adherence to CPG on acute BB-related adverse events.

## Methods

### Study Population

This study retrospectively recruited Chinese patients with HFrEF using electronic medical records (EMRs) from the West China Hospital of Sichuan University (Zhou et al., 2021a) who 1) were discharged between January 1, 2011 and September 30, 2018; 2) were ≥18 years old; 3) had an LVEF <40% (echocardiography reading); 4) had a length of stay >2 days; 5) initiated BBs during hospitalization (did not receive BBs within the two calendar days after admission but a new BB on the third calendar day or later); and 6) had essential records of laboratory tests [serum creatinine, N-terminal pro-B type natriuretic peptide (NT-proBNP), low-density lipoprotein (LDL-C), hemoglobin and cardiac troponin T (cTNT)] and vital signs [heart rate, and systolic and diastolic blood pressures (BPs)]. We excluded patients underwent any non-interventional surgery.

This study was approved by the ethical committee of West China Hospital, Sichuan University (No. 2019-472). The patient consent was waived for data collection of this study was based on the EMR system retrospectively.

### Data Collection

We collected the information of age, sex, smoking, alcohol consumption, surgery, prescription, admission department, discharging diagnoses with International Classification of Diseases, 10th Revision (ICD-10 codes), and patient status on discharge in the EMR system for each patient. We also extracted vital signs (systolic and diastolic BPs, heart rate, and respiration rate), height, and weight from the nursing system, laboratory test results from the laboratory information system (LIS), and LVEF from the echocardiography reading. For patients admitted more than once, we collected the data derived from their first hospitalization. Body mass index (BMI) was calculated as weight (kg) divided by height (m) squared. We used records of prescriptions within the two calendar days after admission, the first record of each vital sign, the first laboratory test results during hospitalization as baseline characteristics for each patient. The estimated glomerular rate filtration (eGFR) was calculated according to the chronic kidney disease epidemiology collaboration formula (Levey et al., 2009; De Cosmo et al., 2016) using age, sex, and serum creatinine values. Hypertension, diabetes mellitus, coronary heart disease (CHD), arrhythmia, cardiomyopathy, rheumatic heart disease, and acute myocardial infarction were identified using the discharging ICD-10 codes I10 to I15, E10 to E14, I20 to I25, I44 to I49, I42, I01 to I09, and I21 to I23, respectively. We calculated the Charlson comorbidity index (CCI) using the ICD-10 codes of discharging diagnoses (Charlson et al., 1987; Fraccaro et al., 2016).

### Initiating Dose of BBs

A standardized initiating dose for each patient was calculated as his/her original initiating dose recorded in the EMR divided by the BB target dose. Recent CPGs recommend that BBs should be initiated at a dose of no more than 1/8 of the target dose (Chinese Society of Cardiology of Chinese Medical Association, 2014; Ponikowski et al., 2016). Patients were identified to be non-adherent to the CPG if they had a standardized initiating dose >1/8, and otherwise in the adherence group.

### Outcomes and Follow up

Time to event outcomes included profound bradycardia (defined as the first episode of heart rate <50 beats per minute during BB administration), hypotension (defined as the first episode of systolic BP <90 mmHg during BB administration), cardiogenic shock requiring intravenous inotropes (defined as the first episode of receiving intravenous milrinone, dobutamine or noradrenaline during BB administration), severe bronchospasm requiring intravenous steroid (defined as the first episode of receiving intravenous methylprednisolone during BB administration), and dose reduction or withdrawal [defined as the first episode of either receiving a standard dose of BB that was less than that of the previous day (reduction) or stopping BB during hospitalization (withdrawal) for any reason]. All patients were followed up throughout the hospitalization. The survival time of a given time-to-event outcome started on the date of the initiating BB dose and ended at the occurrence of this outcome. Patients were considered as censored if they did not have a given outcome before the earlier date of the last BB dose or 7 days after initiation of BBs.

### Statistical Analyses

Descriptive statistics were used for baseline patient characteristics. The normality of variables was tested using the Kolmogorov-Smirnov test. Normal continuous variables were described as mean ± standard deviation (SD) and compared between groups using the two-sided student’s *t*-test. Non-normal continuous variables were shown as median (25% quantile–75% quantile) and compared using the Mann–Whitney *U* test. Categorical variables were reported as frequencies (percentages) and compared using the Chi-square test.

We adopted inverse probability weighting (IPW) to minimize the bias due to potential confounders between two groups. (Cole and Hernán, 2008) We derived propensity score (PS, the estimated probability of initiating BBs with a dose >1/8 of the target dose) for each patient using a multivariable logistic regression model, adjusting for age, sex, baseline heart rate, baseline systolic BP, baseline NT-proBNP, baseline LVEF, baseline eGFR, and CCI. We used estimated PSs to calculate stabilized inverse probability weights which were used in the following analyses to weight the individual contribution to models or cumulative probability (Cole and Hernán, 2008). We presented the effective sample size and the between-group difference of PS before and after applying IPW. (Supplementary Figure S1; Supplementary Table S1 **)** Absolute standardized differences before and after applying IPW were calculated. (Supplementary Figure S2 **)** The baseline characteristics were considered comparable if the absolute standardized difference was less than 0.10. (Supplementary Figure S2).

The IPW-adjusted cumulative incidence was constructed for all outcomes. A Cox proportional hazards regression model was used to assess the association of each time-to-event outcome with guideline non-adherence, weighted by the inverse probability weights. The proportional hazards assumption was evaluated by the proportional risk assumption test. The hazard ratios (HRs) and 95% confidence intervals (CIs) of non-adherence were reported. For data that did not satisfy the assumption of proportional hazards (global *P* < 0.05), we utilized an accelerated failure time model with Weibull distribution to explore the HR and 95% CI of the non-adherence group, adjusting for age, sex, baseline heart rate, baseline systolic BP, baseline NT-proBNP, baseline LVEF, baseline eGFR, and CCI (Lee and Go, 1997).

Patients were stratified by sex (female/male), age (>/≤60 years of age), CHD (with/without), and hypertension (with/without) for subgroup analyses.

We also conducted two sensitivity analyses. The first was to check whether the results were robust to the addition of additional covariates, whether use of CCB, ARB, and venous furosemide within the two calendar days after admission. The performance of IPW and absolute standardized differences between two groups before and after applying IPW were presented in Supplementary Figures S3, S4. The second sensitivity analyses adjusted existed covariates, department of admission (cardiology vs others) and whether the use of oral thiazide at baseline in the generation of the inverse probability weights and multivariable accelerated failure time models. Subgroup analyses included sex, age, status of hypertension, status of CHD, and individual BB (metoprolol succinate vs bisoprolol). The performance of IPW and absolute standardized differences between two groups before and after applying IPW were presented in Supplementary Figures S5, S6.

All analyses were conducted using R Studio (R Pack Version 4.1.0, R Core Team, 2021, R Foundation for Statistical Computing, Vienna, Austria) (R Core Team, 2021), and figures were produced using the package *ggplot2* (Wickham, 2016), *survival* (Therneau T, 2020), and *forestplot* (Max Gordon and Thomas Lumley, 2020). A two-sided *P* value < 0.05 was considered statistically significant.

## Results

### Guideline Adherence of Initiating BBs in Hospitalized patients With HFrEF

**FIGURE 1:**
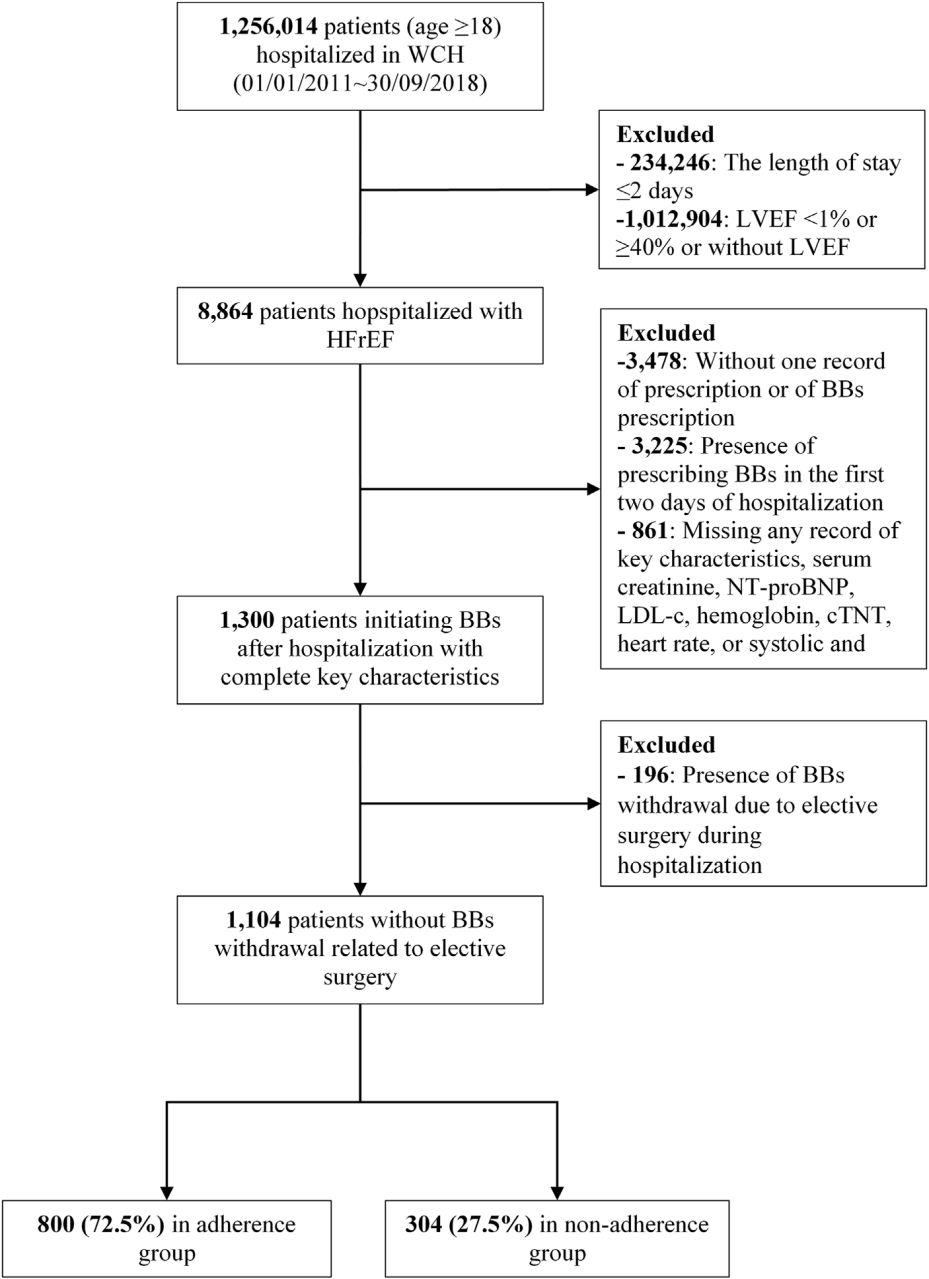
Patient disposition.; Abbreviations: WCH, the West China Hospital of Sichuan University; BB, beta blocker; LVEF, left ventricular ejection factor; NT-proBNP, N-terminal pro-B type natriuretic peptide; LDL-c, low-density lipoprotein; cTNT, cardiac troponin T; HFrEF, heart failure with reduced ejection fraction.

Among 8,864 patients with LVEF <40%, this study included 1,104 HFrEF inpatients who initiated BBs during hospitalization (Figure 1). The baseline characteristics of the patients are shown in Table 1. Among the included patients, 356 (32.2%) patients were female; 633 (57.3%) patients were aged >60 years; 525 (47.6%) patients had CHD; and 446 (40.4%) patients had hypertension at baseline. The median length of hospitalization was 12 days [interquartile range (IQR), 8–17 days].

**TABLE 1:** Baseline patient demographic and clinical characteristics.

Variables	Overall N = 1,104	Adherence group N = 800 (72.5%)	Non-adherence group N = 304 (27.5%)	*P* Value
Age, years	61.2 ± 15.2	62.2 ± 14.9	58.6 ± 15.6	<0.001
BMI, kg/m^2^ ^a^	23.0 (20.8, 25.4)	22.8 (20.6, 25.4)	23.3 (21.3, 25.4)	0.41
LVEF (%)^a^	32.0 (27.0, 36.0)	32.0 (26.0, 36.0)	32.0 (28.0, 36.0)	0.16
Sex, female, *n* (%)	356 (32.2%)	264 (33.0%)	92 (30.3%)	0.38
Smoking, *n* (%)	576 (52.2%)	417 (52.1%)	159 (52.3%)	0.96
Alcohol use, *n* (%)	429 (38.9%)	309 (38.6%)	120 (39.5%)	0.80
CCI^a^	2.0 (1.0, 3.0)	2.0 (1.0, 3.0)	2.0 (1.0, 3.0)	>0.99
Hypertension, *n* (%)	446 (40.4%)	319 (39.9%)	127 (41.8%)	0.57
Diabetes, *n* (%)	298 (27.0%)	220 (27.5%)	78 (25.7%)	0.54
CHD, *n* (%)	525 (47.6%)	408 (51.0%)	117 (38.5%)	<0.001
Acute myocardial infarction, *n* (%)	193 (17.5%)	163 (20.4%)	30 (9.9%)	<0.001
Cardiomyopathy, *n* (%)	393 (35.6%)	285 (35.6%)	108 (35.5%)	0.98
Rheumatic heart disease, *n* (%)	71 (6.4%)	51 (6.4%)	20 (6.6%)	0.90
Arrhythmia, *n* (%)	521 (47.2%)	366 (45.8%)	155 (51.0%)	0.12
Death during hospitalization, *n* (%)	27 (2.4%)	20 (2.5%)	7 (2.3%)	0.85
Admission departments				<0.001
Cardiology, *n* (%)	761 (68.9%)	585 (73.1%)	176 (57.9%)	
Cardiac surgery, *n* (%)	26 (2.4%)	14 (1.8%)	12 (3.9%)	
Nephrology, *n* (%)	22 (2.0%)	10 (1.2%)	12 (3.9%)	
Others, *n* (%)	295 (26.7%)	191 (23.9%)	104 (34.2%)	
HR, beats/minute^a^	88.0 (76.0, 103.0)	89.0 (77.0, 103.0)	85.0 (76.0, 101.2)	0.08
Systolic BP, mmHg^a^	120.0 (105.0, 135.0)	120.0 (104.8, 133.0)	121.0 (108.8, 138.2)	0.03
Diastolic BP, mmHg^a^	76.0 (67.0, 86.0)	75.0 (67.0, 86.0)	78.0 (67.0, 88.0)	0.08
LDL-c, mmol/L^a^	2.2 (1.7, 2.8)	2.2 (1.7, 2.8)	2.3 (1.7, 2.8)	0.42
HbA1c, %^a^	6.4 (5.9, 7.6)	6.4 (5.9, 7.6)	6.4 (5.8, 7.6)	0.26
eGFR, mL/min·1.73 m^2^ ^a^	71.7 (51.6, 92.0)	70.6 (50.6, 91.3)	74.6 (55.6, 94.9)	0.04
NT-proBNP, pg/mL^a^	3,996.0 (1,829.8, 8,331.2)	4,198.0 (1,876.0, 9,140.2)	3,506.5 (1,618.2, 6,802.5)	0.01
ALT, mU/L^a^	29.0 (18.0, 54.0)	30.0 (18.0, 55.0)	27.0 (17.0, 48.5)	0.16
Hb, g/L^a^	133.0 (119.0, 146.0)	132.0 (118.0, 146.0)	134.0 (123.0, 146.0)	0.27
CRP, mg/L^a^	10.6 (4.2, 29.5)	11.1 (4.3, 29.1)	10.4 (3.9, 29.6)	0.37
cTnT, ng/L^a^	38.4 (20.7, 143.4)	42.1 (21.3, 198.0)	33.1 (18.9, 74.0)	<0.001
Standardized initiating dose, ×target dose^a^	0.125 (0.0625, 0.25)	0.125 (0.0625, 0.125)	0.25 (0.25, 0.25)	<0.001
Individual beta blocker				<0.001
Metoprolol succinate, *n* (%)	789 (71.5%)	638 (79.8%)	151 (49.7%)	
Metoprolol tartrate, *n* (%)	72 (6.5%)	66 (8.2%)	6 (2.0%)	
Bisoprolol, *n* (%)	239 (21.6%)	92 (11.5%)	147 (48.4%)	
Others, *n* (%)	4 (0.4%)	4 (0.5%)	0 (0.0%)	
Antihypertensive drugs				
ACEI, *n* (%)	507 (45.9%)	381 (47.6%)	126 (41.4%)	0.07
ARB, *n* (%)	350 (31.7%)	237 (29.6%)	113 (37.2%)	0.02
CCB, *n* (%)	219 (19.8%)	141 (17.6%)	78 (25.7%)	0.003
Diuretic				
Oral furosemide, *n* (%)	335 (30.3%)	263 (32.9%)	72 (23.7%)	0.003
Venous furosemide,*n* (%)	764 (69.2%)	569 (71.1%)	195 (64.1%)	0.03
Oral thiazide, *n* (%)	212 (19.2%)	139 (17.4%)	73 (24.0%)	0.01
Aldosterone receptor antagonist, *n* (%)	932 (84.4%)	692 (86.5%)	240 (78.9%)	0.002

a Data are presented as median (interquartile range) unless otherwise stated.

Abbreviations: ACEI, angiotensin-converting enzyme inhibitor; ALT, alanine aminotransferase; Hb, hemoglobin; ARB, angiotensin II, receptor blockers; BMI, body mass index; NT-proBNP, N-terminal pro-B-type natriuretic peptide; CCB, calcium channel blocker; CCI, Charlson comorbidity Index; CHD, coronary heart disease; CRP, C-reactive protein; cTnT, cardiac troponin T; BP, blood pressure; eGFR, estimated glomerular filtration rate; HbA1c, hemoglobin A1c; HR, heart rate; LDL-c, low-density lipoprotein; LVEF, left ventricular ejection fraction.

The clinicians prescribed BB with a higher dose than the guideline recommendation in 304 (27.5%) patients (median: 1/4 of the target dose). Most patients were initiated BBs within the first few calendar days of admission (Supplementary Figure S7), with a higher portion in non-adherent group (Supplementary Figure S8). Patients in the non-adherence group received more prescriptions of bisoprolol (48.4 vs 11.5%), but less metoprolol succinate (49.7 vs 79.8%) than the adherence group.

Patients in the non-adherence group were with younger age (58.6 ± 15.6 years vs 62.2 ± 14.9 years, *P* < 0.001), less baseline CHD (38.5 vs 51.0%, *P* < 0.001), less baseline acute myocardial infarction (9.9 vs 20.4%, *P* < 0.001); lower NT-proBNP level (median, 3,506.5 pg/ml vs 4,198.0 pg/ml; *P* = 0.01); more prescriptions of ARB or CCB (37.2 vs 29.6%, *P* = 0.02; 25.7 vs 17.6%, *P* = 0.003), and less prescriptions of oral furosemide, venous furosemide, or aldosterone receptor antagonist (23.7 VS 32.9%, *P* = 0.003; 64.1 vs 71.1%, *P* = 0.03; 78.9 vs 86.5%, *P* = 0.002) than the adherence group.

### Guideline Non-Adherence and Adverse Events

**FIGURE 2:**
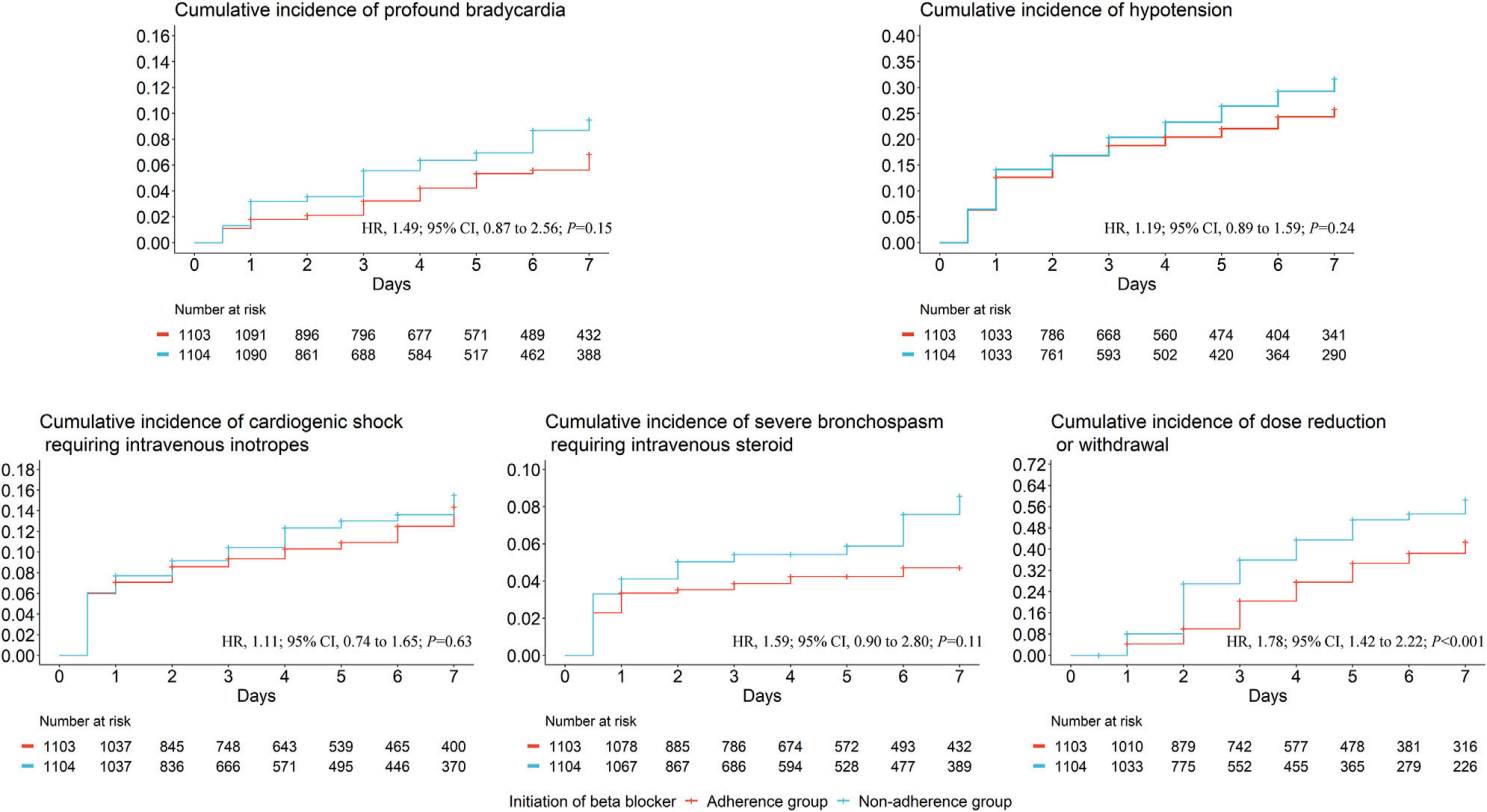
The IPW-adjusted cumulative incidence of each time-to-event adverse events. We derived propensity score for each patient using a multivariable logistic regression model, adjusting for age, sex, baseline heart rate, baseline systolic blood pressure, baseline N-terminal pro-B type natriuretic peptide, baseline left ventricular ejection factor, baseline estimated glomerular filtration rate, and Charlson Comorbidity Index.; Abbreviations: IPW, inverse probability weighting; HR, hazard ratio; CI, confidence interval. Days are calculated from the date of initiating beta blockers.

After applying IPW, baseline characteristics were well balanced between groups. (Supplementary Figure S2) As shown in Figure 2, guideline non-adherence was not significantly associated with a higher risk of profound bradycardia (HR, 1.49; 95% CI, 0.87 to 2.56; *P* = 0.15), hypotension (HR, 1.19; 95% CI, 0.89 to 1.59; *P* = 0.24), cardiogenic shock requiring intravenous inotropes (HR, 1.11; 95% CI, 0.74 to 1.65; *P* = 0.63), and severe bronchospasm requiring intravenous steroid (HR, 1.59; 95% CI, 0.90 to 2.80; *P* = 0.11), but was significantly related to a higher risk of BB dose reduction or withdrawal (HR, 1.78; 95% CI, 1.42 to 2.22; *P* < 0.001).

**FIGURE 3:**
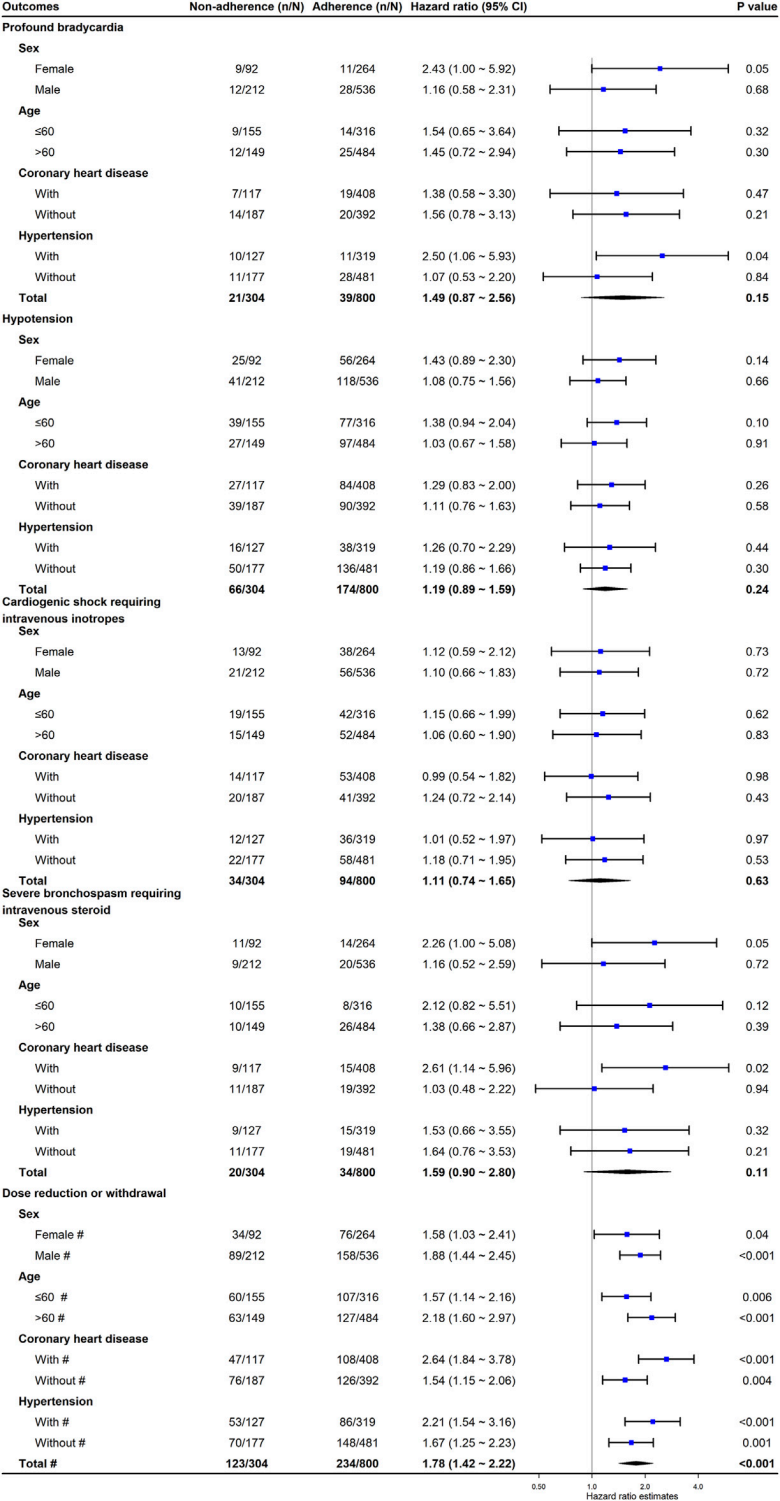
The association between non-adherence to clinical practice guideline recommendations for BB initiation and the risk of adverse events. We derived propensity score for each patient using a multivariable logistic regression model, adjusting for age, sex, baseline heart rate, baseline systolic blood pressure, baseline N-terminal pro-B type natriuretic peptide, baseline left ventricular ejection factor, baseline estimated glomerular filtration rate, and Charlson Comorbidity Index. ^#^ These hazard ratios are derived from accelerate failure time model with Weibull distribution; others are derived from Cox proportional hazards regression model with inverse probability weighting. * The *P* interaction <0.05. Abbreviations: CI, confidence interval; N, number of patients in each group; *n*, number of events in each group.

However, we did not find any subgroup effects by age, sex, with/without CHD, or with/without hypertension on the association of non-adherence to adverse events (all *P* interaction>0.05) (Figure 3).

The first sensitivity analyses showed the results were robust to the addition of additional covariates, whether use of CCB, ARB, and venous furosemide within the two calendar days after admission. (Supplementary Figures S9, S10). The second sensitivity analyses confirmed the robustness of the results and did not find any subgroup effect by individual BB. (Supplementary Figure S11).

## Discussion

This is the first study identifying the guideline adherence of BB initiation in Chinese patients with HFrEF during hospitalization (Chinese Society of Cardiology of Chinese Medical Association, 2014; Ponikowski et al., 2016). Our study shows that non-adherence to BB initiating dose was common in real-world practice. This non-adherent strategy is associated with dose reduction or withdrawal of the drug, which represents the intolerance of the drug or fluctuation of the disease. Although higher dose initiation may not increase the risks of cardiogenic shock requiring intravenous inotropes or severe bronchospasm requiring intravenous steroids during hospitalization, initiating BB adhering to the guideline may fit Chinese patients hospitalized with HFrEF and enhance the confidence and acceptance of the drug.

In patients with newly diagnosed HF during hospitalization, BBs are generally started later than ACEI or ARBs, when the patient is euvolemic, usually shortly before discharge. Particular caution is indicated in patients who have required inotropes during their hospitalization (Ventura, 2004). Our study showed that around a fourth of the patients in our study received a higher dose of BB than recommended by clinical practice guidelines and that over a third of patients reduced or stopped their BBs during hospitalization, which is not commonly supposed in patients with HFrEF. The dose reduction or withdrawal represents the intolerance to the drug due to any reasons, other changes of the patient condition such as worsened cardiac function or unexpected low heart rate. The specific adverse outcomes in the studies could be among those reasons of dose reduction and drug withdrawal of BB, while most causes could link to unpleasant experience and be unable to identify this study. Such unpleasure may shift away the confidence and acceptance of BB use in patients. Such confidence and acceptance are essential to keep people on the drug after discharge. Furthermore, our findings also indicated that high-dose initiation may not end up a high discharging dose of BB due to high rate of dose reduction or discontinuation. Therefore, the dissemination, implementation and adherence to guidelines are critical and relevant to everyday patients in practice and warrants systematic planning and audit (Jin et al., 2021; Wilkinson et al., 2021).

It is worth noting that this proportion of non-adherence is not unique to the practice in China as a similar proportion of non-adherence to guidelines has been reported in primary care in the United Kingdom (Kalra et al., 2013). Taking both these findings together, there is an argument that there is a need to monitor this in wider range of real-world setting. Our study should not dissuade but rather encourage GPs to uptitrate BBs to their target dose given the safety and effectiveness of BBs in HFrEF (Fiuzat et al., 2016; Ajam et al., 2018; Santema et al., 2019). There is an urgent need for a high-quality long-term management solution for Chinese patients with HFrEF with plans for up-titration of BBs in their community rather than the short-term dose achievement of BBs while in hospital following admission with HF.

Current findings show the extent to which the effects of higher initiating BB doses than the guideline recommendation was heterogeneous between sexes, in that the optimal dose of BBs for the treatment of HFrEF may be lower for females than males, which is in line with a report by Santema et al. (2019); this difference might be explained by sex-related differences in pharmacokinetics and pharmacodynamics (Soldin and Mattison, 2009).

The deduction of our finding was limited for its single-center nature and lacked a long-term follow-up. It is also worth noting that there are ethnic differences in responses to BB with previous reports that the Chinese are more sensitive to BB when compared to Caucasians (Zhou et al., 1989; Zhou et al., 2021b). It thus needs external validation in other centers with long-term follow-up and post-discharge safety outcomes to better understand the clinical implications of initial higher doses of BBs. Despite adjustment for several clinical variables, the retrospective study design and heterogeneous nature of the study populations may have resulted in unmeasured confounders that were not accounted for in analyses. Additionally, the dose reduction or withdrawal of BBs represents but not equals to the intolerance to the drug or any adverse events leading to discontinuation. Further studies focusing on patient-reported outcomes such as quality of life and heart failure symptom scores are necessary to validate the current findings.

## Conclusion

This study shows that initiating a starting dose of BB that is not adherent to CPG is common in Chinese patients hospitalized with HFrEF, which is associated with a higher risk of dose reduction or withdrawal during hospitalization. Thus, a “start low, go slow” strategy for BB treatment is appropriate to minimize drug tolerance, and the guideline adherent audit is necessary to improve the patients’ outcomes in the real-world hospital setting.

## Data Availability

The original data was not available due to the protection of patients’ privacy. Requests to access the datasets should be directed to lisheyu@gmail.com.

## Abbreviations

ACEI, angiotensin-converting enzyme inhibitor; ALT, alanine aminotransferase; ARB, angiotensin II receptor blockers; BB, beta-blocker; BMI, body mass index; BP, blood pressure; CCB, calcium channel blocker; CCI, Charlson comorbidity Index; CHD, coronary heart disease; CI, confidence interval; CPGs, clinical practice guidelines; CRP, C-reactive protein; cTnT, cardiac troponin T; eGFR, estimated glomerular filtration rate; EMRs, electronic medical records; Hb, hemoglobin; HbA1c, hemoglobin A1c; HF, heart failure; HFrEF, heart failure with reduced ejection fraction; HR, heart rate; IPW, inverse probability weighting; IQR, interquartile range; LDL-c, low-density lipoprotein; LIS, laboratory information system; LVEF, left ventricular ejection fraction; NT-proBNP, N-terminal pro-B-type natriuretic peptide; WCH, West China Hospital of Sichuan University.
