# Supplementary material for: Guideline Adherence of β-blocker Initiating Dose and its Consequence in Hospitalized Patients With Heart Failure With Reduced Ejection Fraction
Source: Front Pharmacol. 2021 Nov 16;12:770239. doi: 10.3389/fphar.2021.770239 (PMC8660072; doi:10.3389/fphar.2021.770239)
Supplement: Supplementary file 1 [file DataSheet1.docx]

Supplementary Material

# Supplementary Figures and Tables

## Supplementary Tables

**Supplementary Table 1.** The effective sample size in the adherence to clinical practice guidelines group and the non-adherence group before and after applying inverse probability weighting.

|  | Before weighting | After weighting |
| --- | --- | --- |
| Adherence group | 800 | 790 |
| Non-adherence group | 304 | 279 |
| Total | 1104 | 1069 |

## Supplementary Figures

**Supplementary Figure 1.** The distribution of propensity score for patients in the adherence to clinical practice guidelines group and the non-adherence group before and after inverse probability weighting.

**
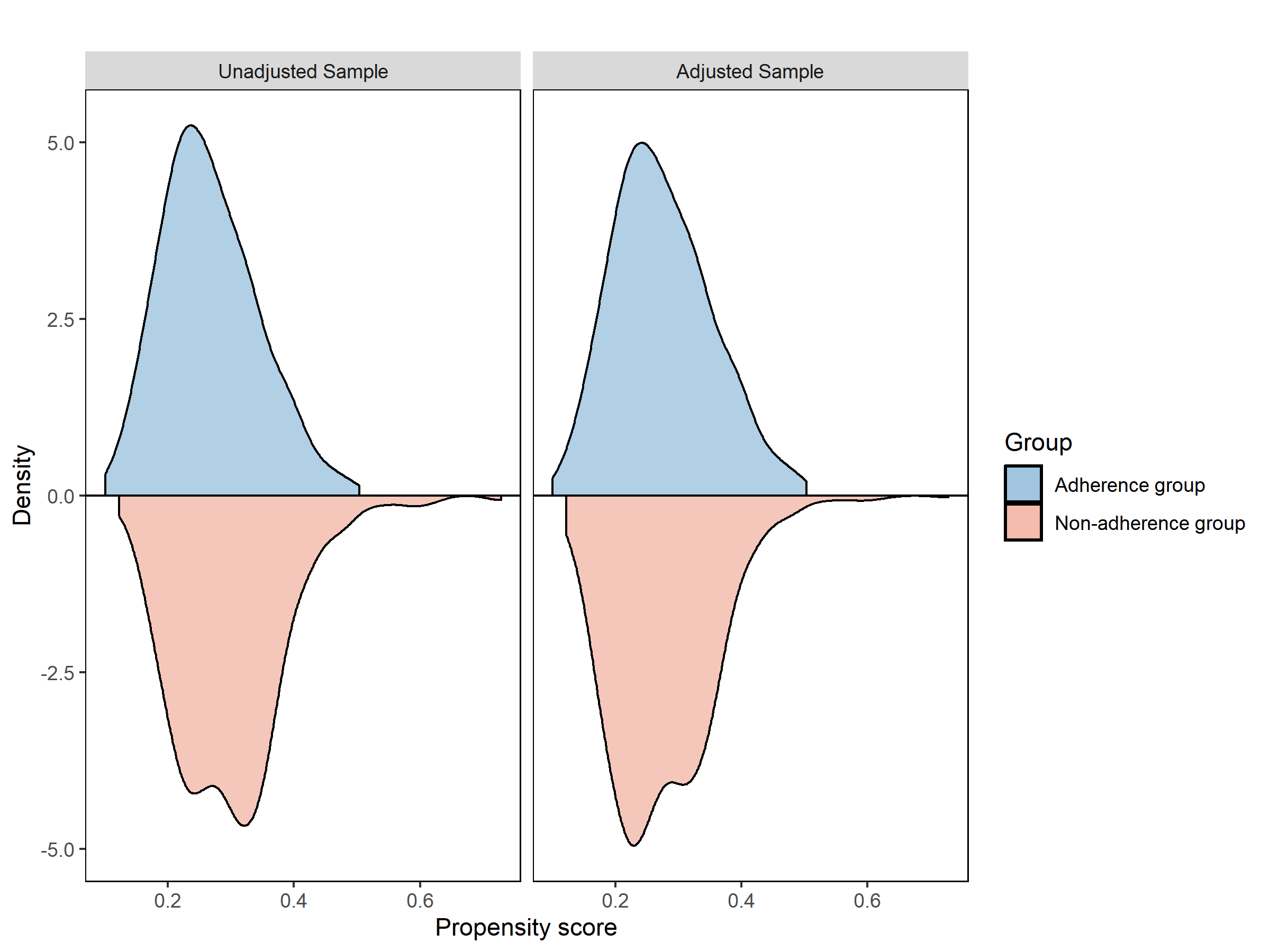
**

We derived propensity score for each patient using a multivariable logistic regression model, adjusting for age, sex, baseline heart rate, baseline systolic blood pressure, baseline N-terminal pro-B type natriuretic peptide, baseline left ventricular ejection factor, baseline estimated glomerular filtration rate, and Charlson Comorbidity Index.

**Supplementary Figure 2.** Balance of covariates across groups before and after inverse probability weighting.


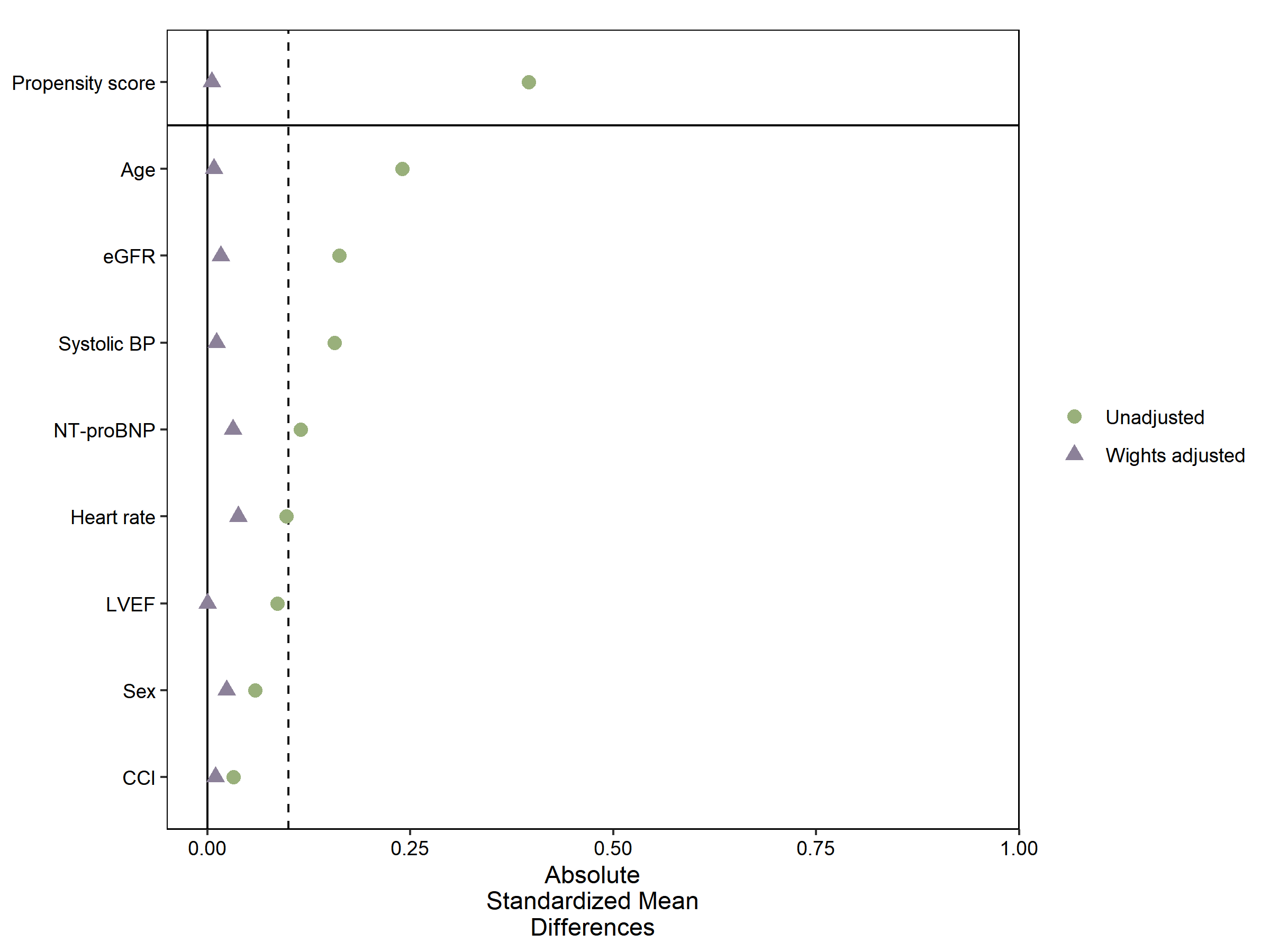


We derived propensity score for each patient using a multivariable logistic regression model, adjusting for age, sex, baseline heart rate, baseline systolic blood pressure, baseline N-terminal pro-B type natriuretic peptide, baseline left ventricular ejection factor, baseline estimated glomerular filtration rate, and Charlson Comorbidity Index.

Abbreviations: BP, blood pressure; NT-proBNP, N-terminal pro-B-type natriuretic peptide; LVEF, left ventricular ejection fraction; CCI, Charlson Comorbidity Index; eGFR, estimated glomerular rate filtration.

**Supplementary Figure 3.** The distribution of propensity score for patients in the adherence to clinical practice guidelines group and the non-adherence group before and after inverse probability weighting when conducting the first sensitivity analyses.

**
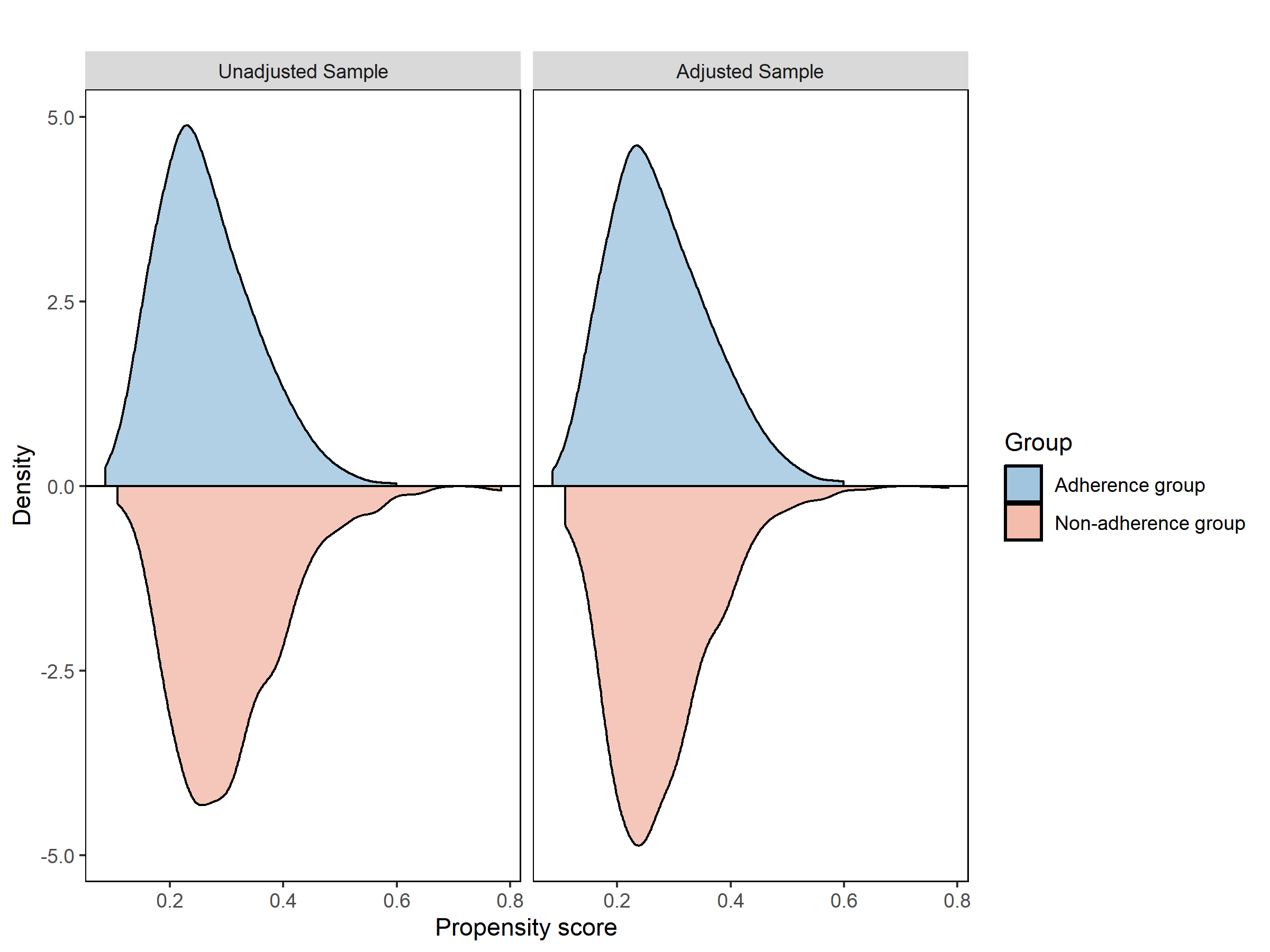
**

In the first sensitivity analyses, we derived propensity score for each patient using a multivariable logistic regression model, adjusting for age, sex, baseline heart rate, baseline systolic blood pressure, baseline N-terminal pro-B type natriuretic peptide, baseline left ventricular ejection factor, baseline estimated glomerular filtration rate, Charlson Comorbidity Index, and use of medications at baseline, including whether use of calcium channel blocker, whether use of angiotensin II receptor blockers, and whether use of venous furosemide.

**Supplementary Figure 4.** Balance of covariates across groups before and after inverse probability weighting when conducting the first sensitivity analyses.


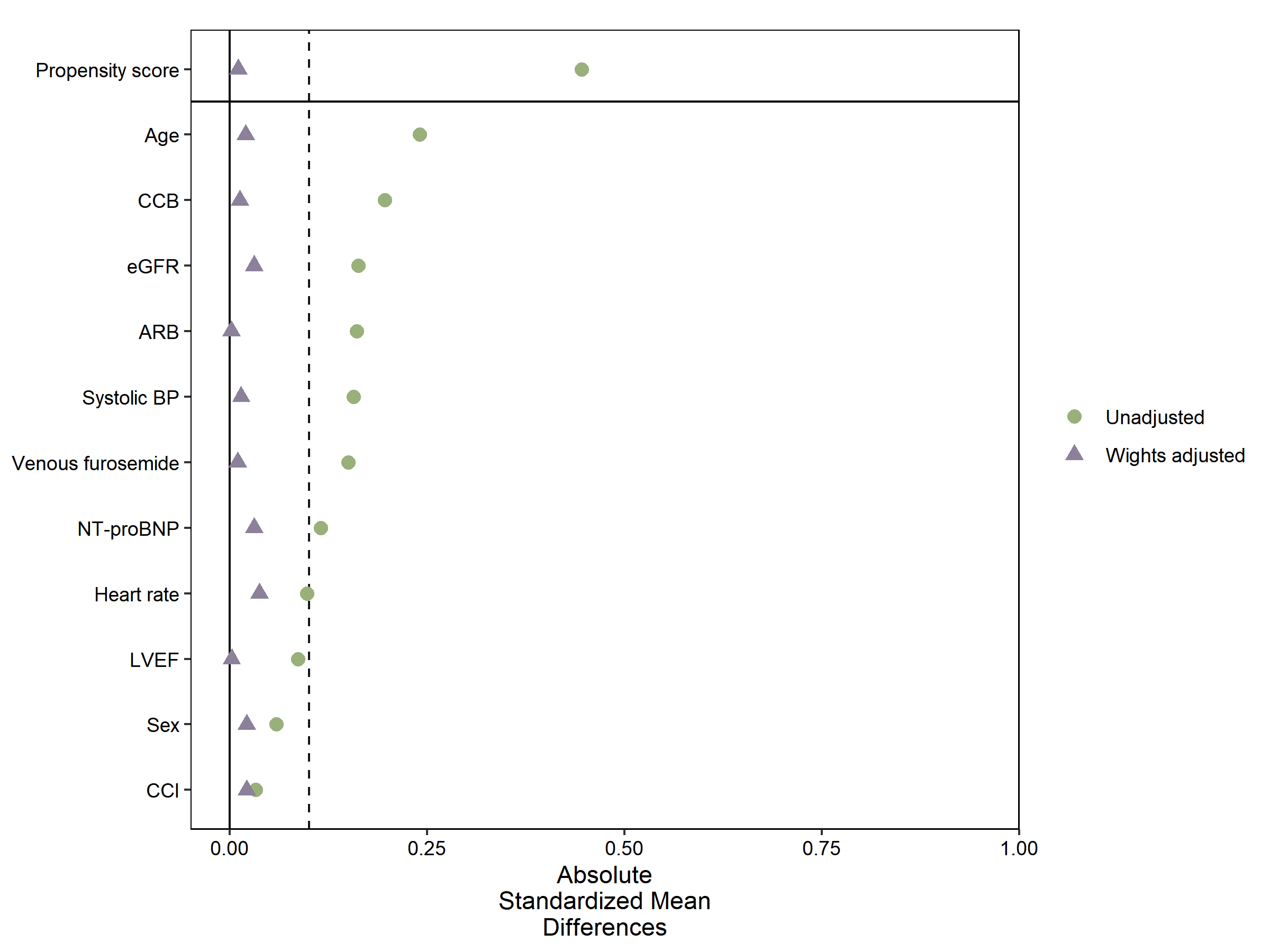


In the first sensitivity analyses, we derived propensity score for each patient using a multivariable logistic regression model, adjusting for age, sex, baseline heart rate, baseline systolic blood pressure, baseline N-terminal pro-B type natriuretic peptide, baseline left ventricular ejection factor, baseline estimated glomerular filtration rate, Charlson Comorbidity Index, and use of medications at baseline, including whether use of calcium channel blocker, whether use of angiotensin II receptor blockers, and whether use of venous furosemide.

Abbreviations: BP, blood pressure; NT-proBNP, N-terminal pro-B-type natriuretic peptide; LVEF, left ventricular ejection fraction; CCI, Charlson Comorbidity Index; eGFR, estimated glomerular rate filtration; CCB, calcium channel blocker; ARB, angiotensin II receptor blockers.

**Supplementary Figure 5.** The distribution of propensity score for patients in the adherence to clinical practice guidelines group and the non-adherence group before and after inverse probability weighting when conducting the second sensitivity analyses.

**
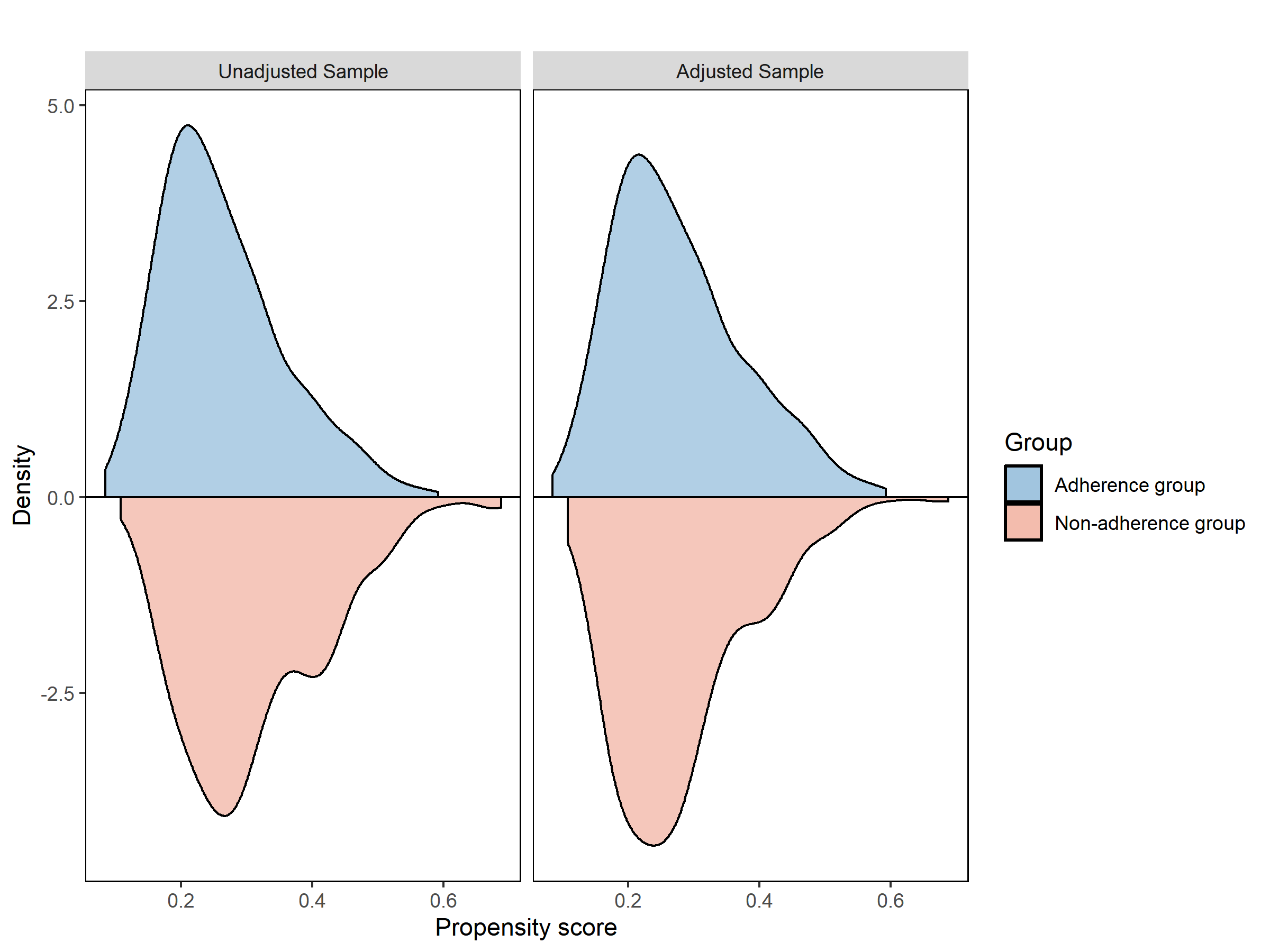
**

In the second sensitivity analyses, we derived propensity score for each patient using a multivariable logistic regression model, adjusting for age, sex, baseline heart rate, baseline systolic blood pressure, baseline N-terminal pro-B type natriuretic peptide, baseline left ventricular ejection factor, baseline estimated glomerular filtration rate, Charlson Comorbidity Index, department of admission (cardiology vs others), and whether use of oral thiazides at baseline.

**Supplementary Figure 6.** Balance of covariates across groups before and after inverse probability weighting when conducting the second sensitivity analyses.


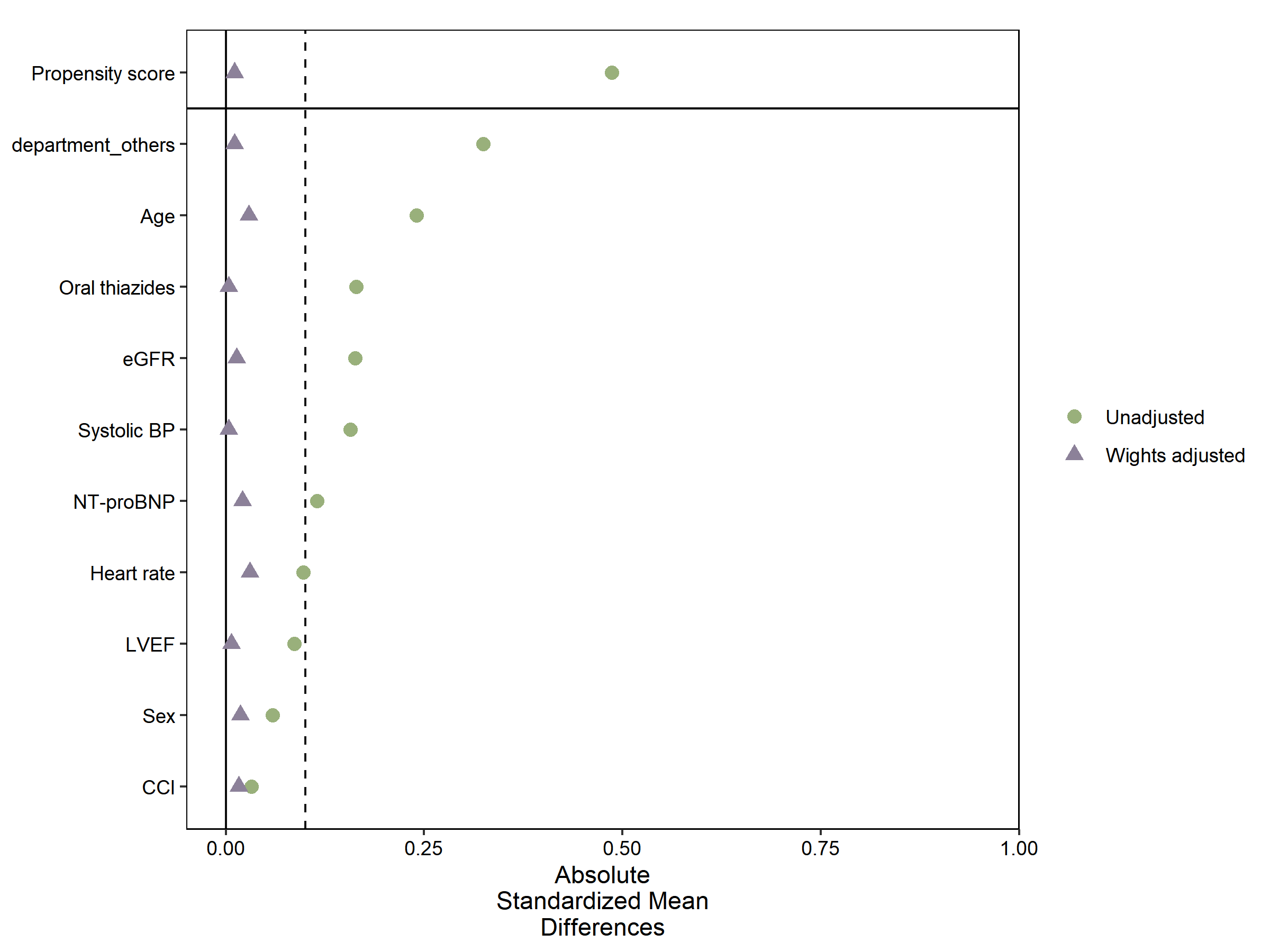


In the second sensitivity analyses, we derived propensity score for each patient using a multivariable logistic regression model, adjusting for age, sex, baseline heart rate, baseline systolic blood pressure, baseline N-terminal pro-B type natriuretic peptide, baseline left ventricular ejection factor, baseline estimated glomerular filtration rate, Charlson Comorbidity Index, department of admission (cardiology vs others), and whether use of oral thiazides at baseline.

Abbreviations: BP, blood pressure; NT-proBNP, N-terminal pro-B-type natriuretic peptide; LVEF, left ventricular ejection fraction; CCI, Charlson Comorbidity Index; eGFR, estimated glomerular rate filtration.

**Supplementary Figure 7.** The distribution of the calendar day of the date when patients were initiated beta blocker during hospitalization from admission


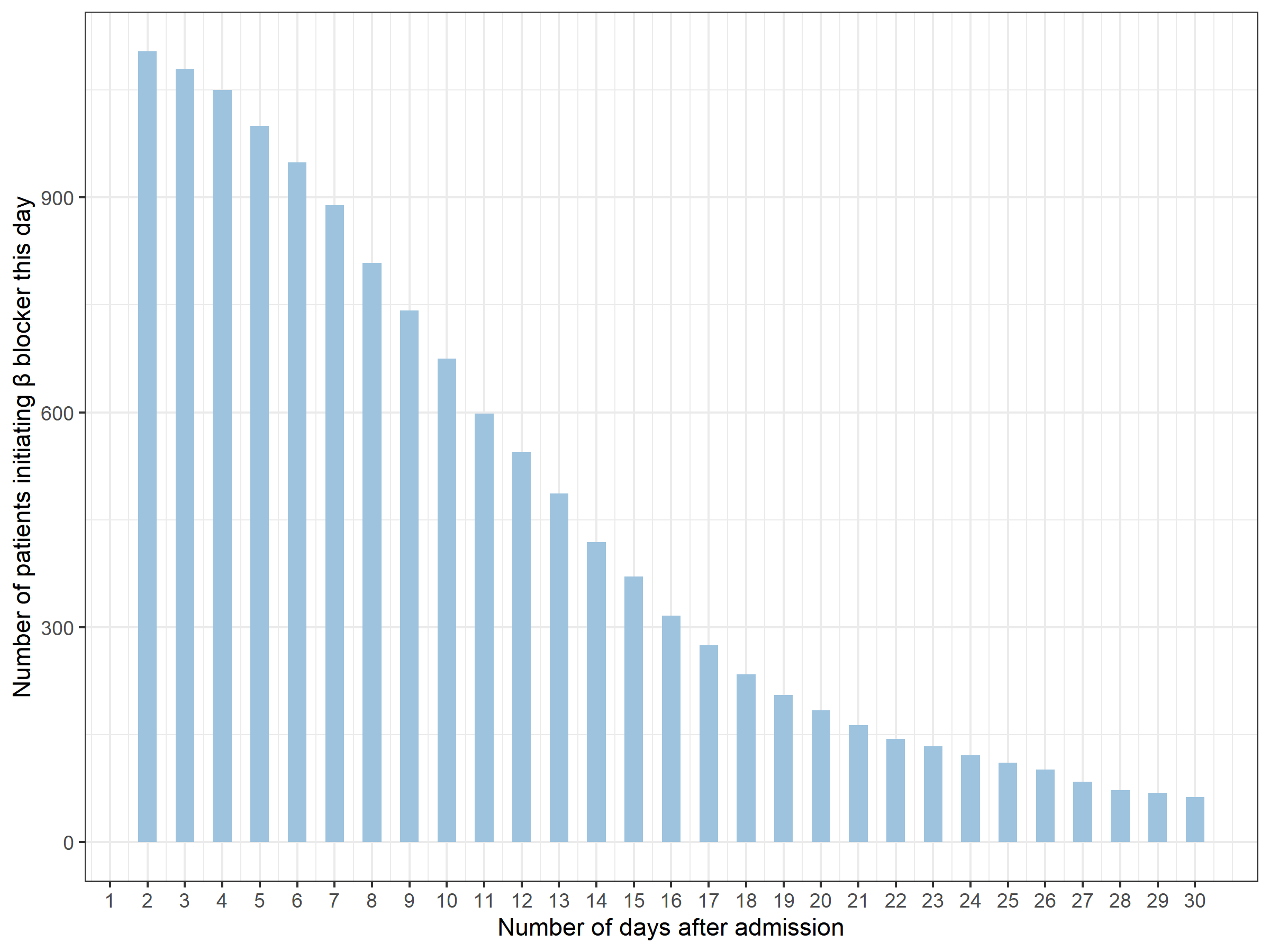


**Supplementary Figure 8.** The comparison of the calendar day of the date when patients were initiated beta blocker during hospitalization from admission between the adherence group and the non-adherence group


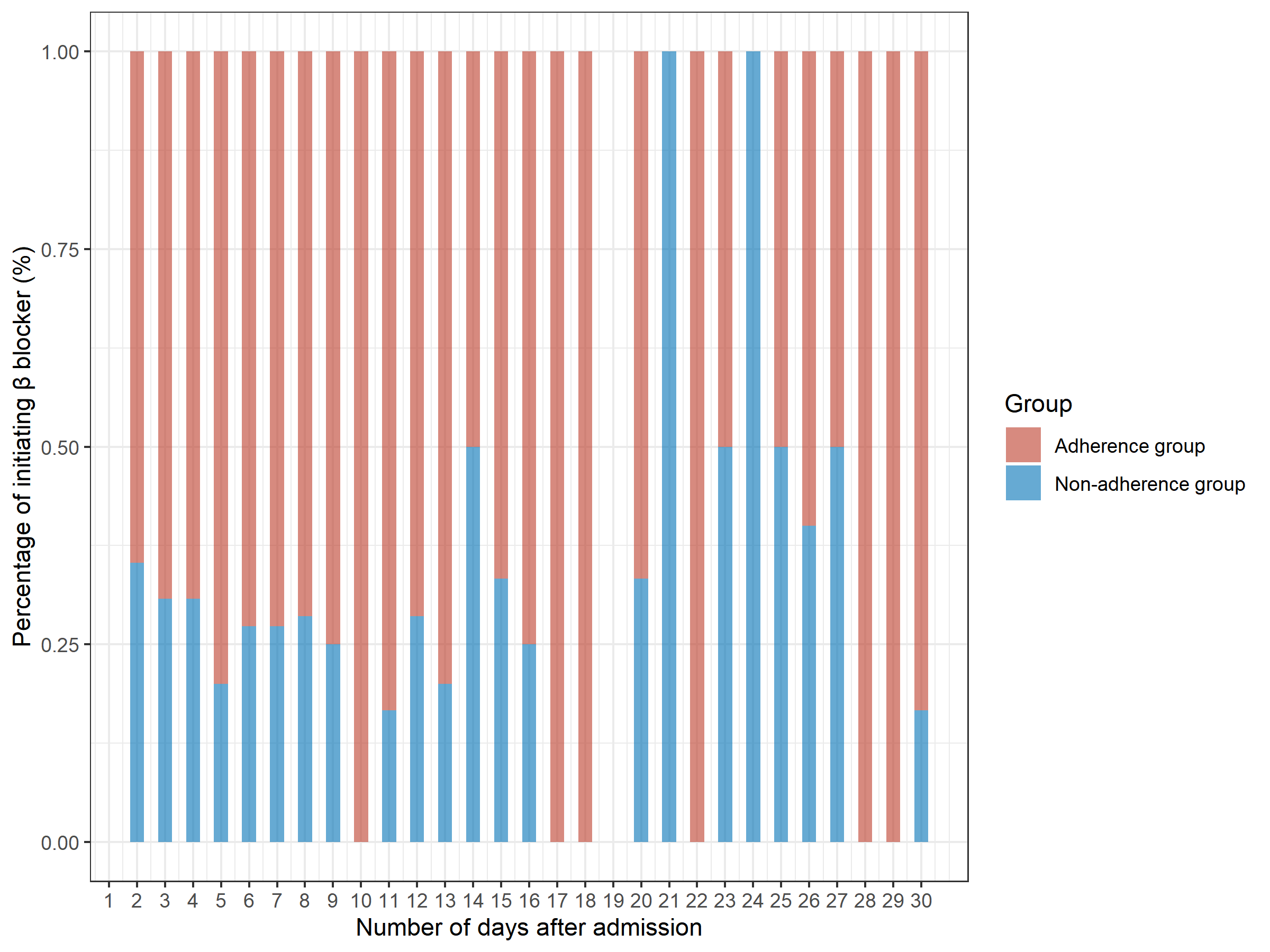


**Supplementary Figure 9.** The IPW-adjusted cumulative incidence of each time-to-event adverse events in the first sensitivity analyses.


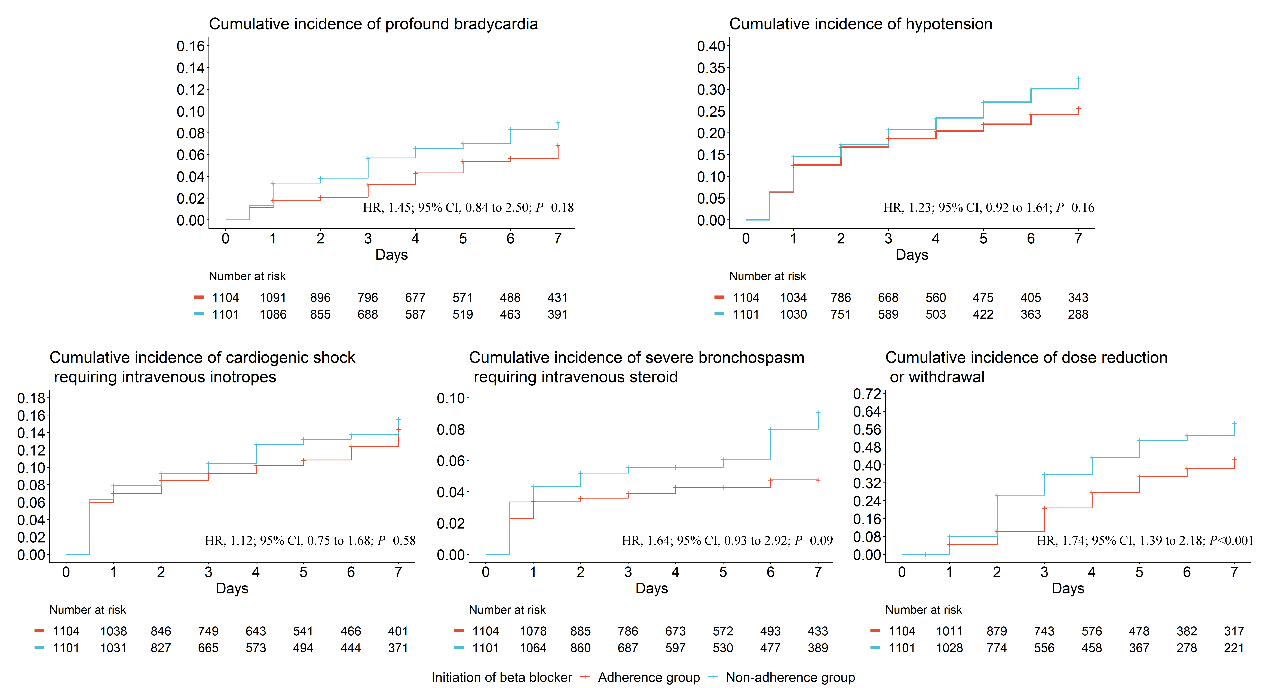


In the first sensitivity analyses, we derived propensity score for each patient using a multivariable logistic regression model, adjusting for age, sex, baseline heart rate, baseline systolic blood pressure, baseline N-terminal pro-B type natriuretic peptide, baseline left ventricular ejection factor, baseline estimated glomerular filtration rate, Charlson Comorbidity Index, and use of medications at baseline, including whether use of calcium channel blocker, whether use of angiotensin II receptor blockers, and whether use of venous furosemide.

Abbreviations: IPW, inverse probability weighting; HR, hazard ratio; CI, confidence interval. Days are calculated from the date of initiating beta blockers.

**Supplementary Figure 10.** The association between non-adherence to clinical practice guideline recommendations for BB initiation and the risk of adverse events in the first sensitivity analyses.


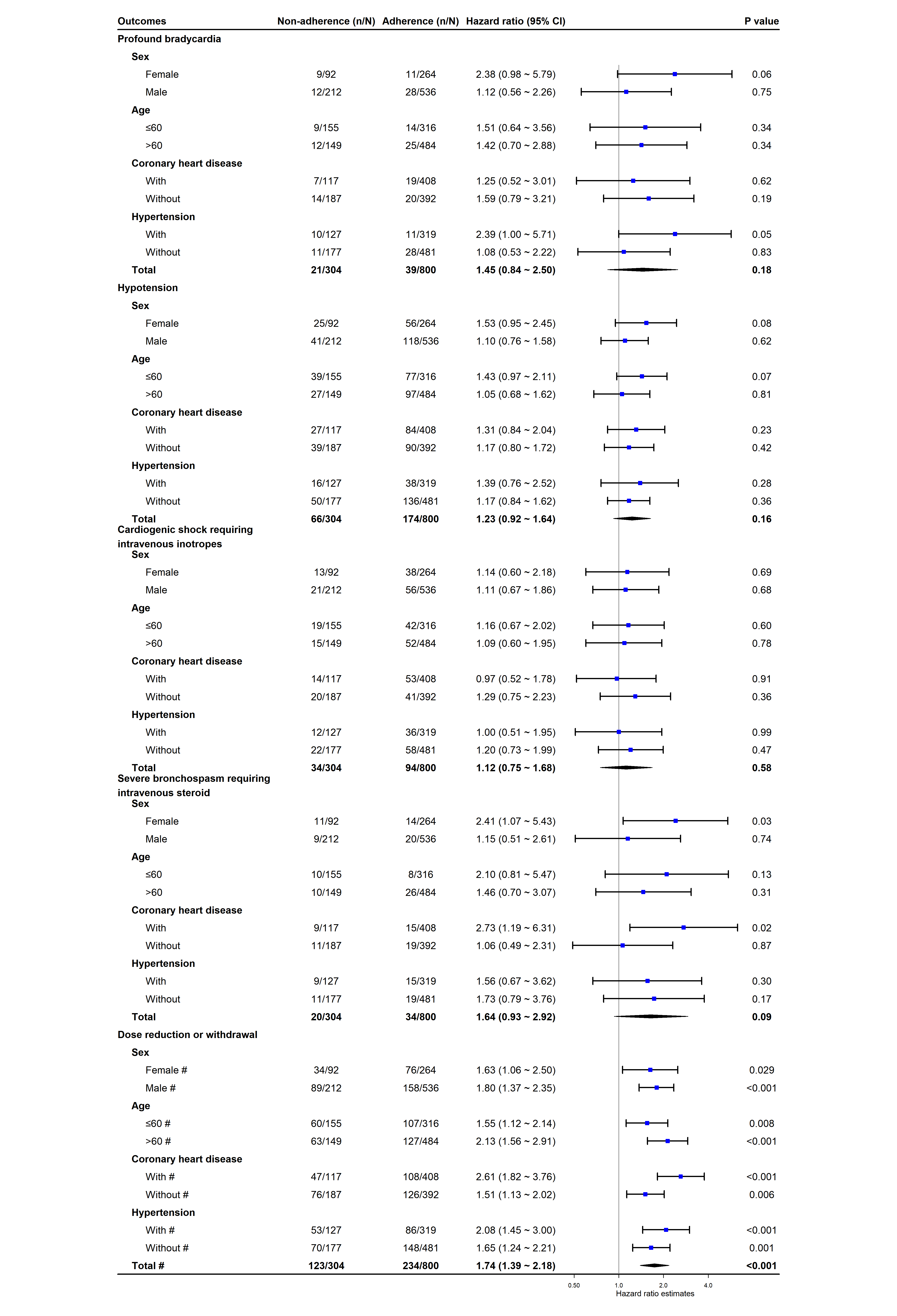


In the first sensitivity analyses, we derived propensity score for each patient using a multivariable logistic regression model, adjusting for age, sex, baseline heart rate, baseline systolic blood pressure, baseline N-terminal pro-B type natriuretic peptide, baseline left ventricular ejection factor, baseline estimated glomerular filtration rate, Charlson Comorbidity Index, and use of medications at baseline, including whether use of calcium channel blocker, whether use of angiotensin II receptor blockers, and whether use of venous furosemide.

^#^ These hazard ratios are derived from accelerate failure time model with Weibull distribution; others are derived from Cox proportional hazards regression model with inverse probability weighting. * The p interaction <0.05.

Abbreviations: CI, confidence interval; N, number of patients in each group; n, number of events in each group.

**Supplementary Figure 11.** The association between non-adherence to clinical practice guideline recommendations for BB initiation and the risk of adverse events in the second sensitivity analyses.


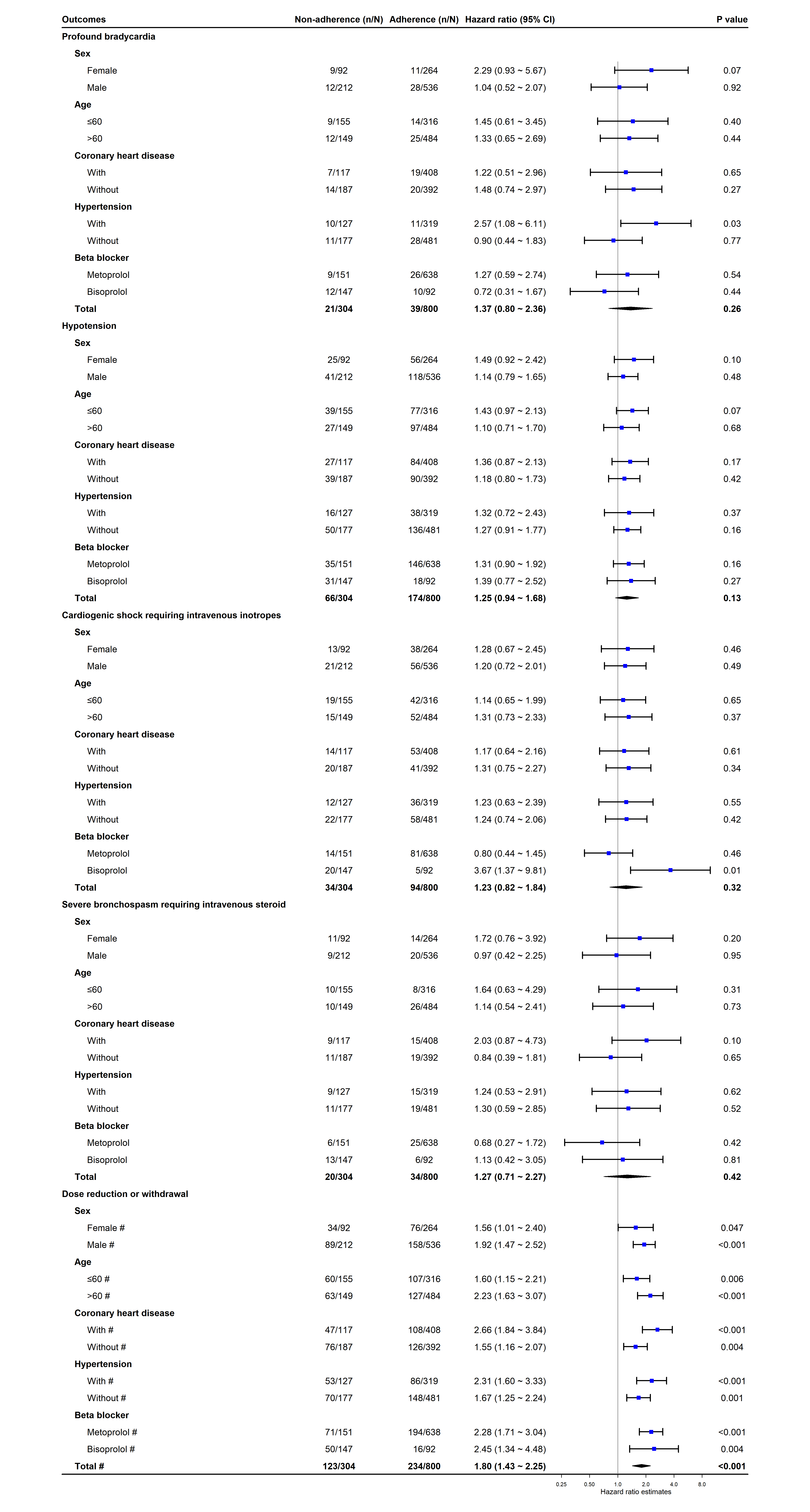


In the second sensitivity analyses, we derived propensity score for each patient using a multivariable logistic regression model, adjusting for age, sex, baseline heart rate, baseline systolic blood pressure, baseline N-terminal pro-B type natriuretic peptide, baseline left ventricular ejection factor, baseline estimated glomerular filtration rate, Charlson Comorbidity Index, department of admission (cardiology vs others), and whether use of oral thiazides at baseline.

^#^ These hazard ratios are derived from accelerate failure time model with Weibull distribution; others are derived from Cox proportional hazards regression model with inverse probability weighting. * The p interaction <0.05.

Abbreviations: CI, confidence interval; N, number of patients in each group; n, number of events in each group.
